# Alterations of Gut Microbiome in Tibetan Patients With Coronary Heart Disease

**DOI:** 10.3389/fcimb.2020.00373

**Published:** 2020-07-23

**Authors:** Fengyun Liu, Chao Fan, Liangzhi Zhang, Yuan Li, Haiwen Hou, Yan Ma, Jinhua Fan, Yueqin Tan, Tianyi Wu, Shangang Jia, Yanming Zhang

**Affiliations:** ^1^National Key Laboratory of High Altitude Medicine, Qinghai High Altitude Medical Research Institute, Xining, China; ^2^Qinghai Province Cardiovascular and Cerebrovascular Disease Specialist Hospital, Xining, China; ^3^Key Laboratory of Adaptation and Evolution of Plateau Biota, Northwest Institute of Plateau Biology, Chinese Academy of Sciences, Xining, China; ^4^University of Chinese Academy of Sciences, Beijing, China; ^5^College of Grassland Science and Technology, China Agricultural University, Beijing, China

**Keywords:** gut microbiome, 16S rDNA, metagenome, coronary heart disease (CHD), Tibetan, lipopolysaccharide (LPS)

## Abstract

Coronary heart disease (CHD) is closely related to gut microbiota, which may be significantly affected by ethnicity and the environment. Knowledge regarding the gut microbiome of Tibetan CHD patients living in the Qinghai–Tibet Plateau is very limited. In this study, we characterized the physiological parameters and gut microbiota from 23 healthy Tibetans (HT), 18 CHD patients, and 12 patients with non-stenosis coronary heart disease (NCHD). We analyzed the alterations of the gut microbiome in CHD patients and investigated the relationship between these alterations and the pathological indicators. We found no changes in trimethylamine N-oxide, however, a significant increase in lipopolysaccharides and white blood cells, and a decrease in high-density lipoprotein were observed in the blood of CHD patients, compared to that in the HT group. The gut microbiota of the NCHD group had a significantly higher Shannon index than that of the HT group. Adonis analysis showed that both microbial compositions and functions of the three groups were significantly separated. The *Dialister* genus was significantly lower and *Blautia, Desulfovibrio*, and *Succinivibrio* were significantly higher in abundance in CHD patients compared with the HT group, and the changes were significantly correlated with physiological indexes, such as increased lipopolysaccharides. Moreover, enrichment of genes decreased in four pathways of amino acid metabolism, such as arginine biosynthesis and histidine metabolism, although two lipid metabolism pathways, including fatty acid degradation and arachidonic acid metabolism, increased in the CHD group. Additionally, occupation and a family history of CHD were shown to be risk factors and affected the gut microbiota in Tibetans. Our study will provide insights into the understanding of CHD, leading to better diagnosis and treatment of Tibetan patients.

## Introduction

Trillions of microbes inhabit the human gut. This microbiome is important for health, acting as a second genome and an additional endocrine organ in humans (Eckburg et al., 2005; Schroeder and Bäckhed, 2016). The gut microbiota provides nutrients and energy to the host through ingested food, and produces metabolic bioactive signaling molecules to maintain health or elicit diseases, such as cardiovascular disease, which is the leading cause of death worldwide (Wang and Zhao, 2018). According to the *China Cardiovascular Diseases Report 2018* (Hu et al., 2018), there were approximately 290 million people with cardiovascular disease in China, with 11 million afflicted with coronary heart disease (CHD).

The gut microbiota appears to differ significantly between patients with CHD and healthy subjects (Emoto et al., 2016, 2017). Gut microbial enzymes that produce trimethylamine (TMA) are more active in patients with coronary artery disease than in healthy individuals (Jie et al., 2017). Dietary phosphatidylcholine is metabolized by intestinal microorganisms into TMA, which is converted into TMA N-oxide (TMAO) in the liver. TMAO enters and accumulates in the peripheral blood, which accelerates the atherosclerosis process and increases the incidence of cardiovascular disease (Wang et al., 2011; Tang et al., 2013). However, a study involving human and mouse samples revealed that *Bacteroides* and multiflora in the intestinal tract may reduce atherosclerosis formation by inhibiting lipopolysaccharide (LPS)-induced inflammation, which may be a new strategy for the prevention and treatment of CHD (Yoshida et al., 2018). Because the gut microbial functions depend on combined host and environmental factors, associated disorders that are common in patients with atherosclerosis remain difficult to understand.

Few studies have examined the correlation between cardiovascular diseases in ethnic populations and their gut microbiome. This lack of knowledge includes the population of Tibetan indigenous people living in the plateau region. Tibetans are a population with unique adaptations to extreme environments. Subjects living on the Qinghai-Tibet Plateau have adjusted to an average altitude exceeding 3,000 m. Animal husbandry provides their main food source. The diet is enriched in red meat and milk, which is very different from the primarily starchy grain diet of the farming population (Deschasaux et al., 2018; He Y. et al., 2018). The gut microbiome of Tibetan individuals is significantly different from that of other populations, such as the Han (Li and Zhao, 2015) and Mongolian populations (Zhang et al., 2015; Liu et al., 2016).

The objective of this study was to investigate alterations of the gut microbiome in Tibetan patients with CHD and their correlations with physiological indicators. Fecal microbiota from 23 healthy Tibetans (HT), 18 Tibetan inpatients with CHD, and 12 Tibetan inpatients with non-stenosis coronary heart disease (NCHD) were profiled by 16S ribosomal DNA (rDNA), and physiological parameters were measured in the three groups. Metagenomic sequencing and Kyoto Encyclopedia of Genes and Genomes (KEGG) functional analysis of 39 samples (12 CHD, 9 NCHD, and 18 HT) were performed. Bacterial community structures, co-occurrence networks, and functional differences between the samples were assessed. We also explored the relationship between the gut microbiome and physiological parameters to better explain the pathogenesis. In addition, we performed a preliminary examination of the effects of occupation and family genetic background on the gut microbiota of Tibetan patients with CHD. Our findings provide insights for the prevention and treatment of CHD in this population.

## Materials and Methods

### Recruitment of Patients and Volunteers

The study was approved on 09 March 2017 by the Ethics Committee of Qinghai Province Cardiovascular and Cerebrovascular Disease Specialist Hospital, China. All subjects provided written informed consent in accordance with the Declaration of Helsinki. Informed consent was obtained after written introductions were provided to all 53 Tibetan volunteers from the Tibetan Autonomous County in the Qinghai Province of China. None of the respondents used antibiotics, probiotics, or prebiotics for at least 3 months before sampling, and their ages ranged from 40 to 70 years.

The diagnostic records of the Tibetan patients were collected from August 2017 to February 2018 from the cardiac center, Qinghai Cardio-cerebrovascular Disease Specialist Hospital in Xining City. Thirty Tibetan patients completed questionnaires that included information. Subjects without CHD and acute and chronic inflammatory diseases that may affect gut microbiota, including diseases of the digestive system, tumors, renal failure, and other serious diseases were excluded.

The 30 Tibetan patients were divided into two groups according to their coronary angiography results. 18 patients with coronary artery stenosis >50% were placed in the CHD group and 12 patients with coronary artery stenosis <50% were placed in the non-stenosis coronary heart disease (NCHD) group. The patients in the NCHD group had coronary risk factors, including hypertension, dyslipidemia, arrhythmia, and ischaemic changes evident on an electrocardiogram (ECG) (Table S1).

Twenty three HTs, most of whom were patients' accompanying nursing professionals, were included in the control group. The individuals in the HT group were family members of the patients and so their diets were similar to the patients. Exclusion criteria for the HT group included those with a history of cardiovascular disease, diabetes, and patients with treated hyperlipidaemia. All participants in the HT group were Tibetan native residents, had similar living habits, and were required to complete the same questionnaires as the patients. The ECG and biochemical indexes in the blood were investigated in the HT group.

### Collection of Stool and Blood Samples

Fecal samples were collected at the hospital while the patients consumed the hospital diet and the samples were immediately frozen at −80°C before being further processed. Next, the samples were submerged in 30 mL of 97% ethanol for 24–36 h. The remaining solid materials were transferred to 50 mL tubes with silica beads (Sigma-Aldrich, St. Louis, MO, USA). Blood samples were collected in the morning following overnight fasting conditions. Plasma was collected by centrifugation and stored at −80°C until use.

### General Pathological Plasma Biochemical Analyses

Biochemical parameters in plasma, renal function parameters, blood lipids, clotting factors, and blood cell counts were determined using a model AU2700 fully automatic blood biochemical analyser (Olympus, Tokyo, Japan). The 17 parameters assessed were: triglyceride (TG), total cholesterol (TC), high-density lipoprotein (HDL), low-density lipoprotein (LDL), apolipoprotein A1 (APO-A1), apolipoprotein B (APO-B), lipoprotein(a) [LP(a)], glucose (GLU), blood urea nitrogen/creatinine (BUN/Cr), uric acid (UA), white blood cell (WBC) count, red blood cell (RBC) count, hemoglobin (HGB), haematocrit (HCT), platelet (PLT) count, homocysteine (Hcy), and C-reactive protein (CRP).

### Determinants of TMAO- and TMAO-Related Metabolites in Plasma

#### Preparation of Standard Solution

Metabolites of choline, betaine, creatinine, and l-carnitine, which are associated with TMAO, were selected for preparation of the standard solution. Choline, betaine, TMAO, creatinine, and l-carnitine were accurately weighed, and a high concentration of the standard solution was prepared as an internal solution with 1% formate-acetonitrile. The standard solution was stored at −20°C for short-term use.

#### Sample Pre-treatment

In 20 μL of the sample or standard solutions, 10 μL of internal standard solution was added, followed by 750 μL of 1% formic acid-acetonitrile solution, mixed, and centrifuged. The supernatant was collected.

#### Liquid Chromatography-Tandem Mass Spectrometry Analysis

A high-performance liquid chromatography-tandem mass spectrometry method was applied to explore the metabolites of choline, TMAO, betaine, creatinine, and l-carnitine in plasma. Chromatographic separation was performed on an ACQUITY UPLC® BEH HILIC 2.1 column (2.1 × 100 mm, 1.7 μM; Waters, Beverly, MA, USA), with 5 μL of the sample, mobile phase A-acetonitrile, B-water, including 10 mm ammonium acetate, and a flow rate of 0.4 mL/min at 40°C. The gradient elution program was as follows: 0~1 min, 80% A; 1~2 min, 80~70% A; 2~2.5 min, 70% A; 2.5~3 min, 70~50% A; 3~3.5 min, 50% A; 3.5~4 min, 50~80% A; and 4~6 min, 80% A. Mass spectrometry elution was performed with an electrospray ionization source in the positive ion mode. Multiple response monitoring scans and quantitative analyses were performed on all plasma samples.

### LPS Levels in Plasma and Feces

A double-antibody one-step sandwich enzyme-linked immunosorbent assay kit was used for LPS evaluation. The samples, standards, and horseradish peroxidase-labeled detection antibodies were loaded into the coated micropores with precoated human LPS-captured antibodies and incubated thoroughly before washing. The 3, 3′, 5, 5′-tetramethylbenzidine (TMB) substrate was converted to blue in the presence of peroxidase and to the final yellow color with the addition of an acid stop solution. To quantify LPS, absorbance was measured on a microplate reader at 450 nm and the sample concentration was calculated by comparison to a standard curve.

### DNA Extraction and Sequencing

DNA from fecal material was extracted using a QIAamp DNA Stool Mini Kit (QIAGEN, Dusseldorf, Germany) following the standard protocol. The DNA concentration was determined using a NanoDrop ND-1000 (Thermo Fisher Scientific, Waltham, MA, USA). The V3 and V4 regions of 16S rDNA from all samples were amplified using the 341F (5′-CCTAYGGGRBGCASCAG-3′) and 806R (5′-GGACTACNNGGGTATCTAAT-3′) primers. The polymerase chain reaction (PCR) products were quantified and purified using a QuantiFluor™ fluorometer (Promega Biotech, Madison, WI, USA). Negative controls included no template controls for DNA extraction and PCR amplification. PCR products were mixed in equidensity ratios. The mixture of PCR products was purified with a Gel Extraction Kit (QIAGEN). Sequencing libraries were generated using the TruSeq® DNA PCR-Free Sample Preparation Kit (Illumina, San Diego, CA, USA) following the manufacturer's recommendations, and index codes were added. The library quality was assessed on a Qubit@ 2.0 Fluorometer (Thermo Fisher Scientific) and the Bioanalyzer 2100 system (Agilent, Santa Clara, CA, USA). Finally, the library was sequenced on a HiSeq2500 platform (Illumina), and 250 bp paired-end reads were generated.

DNA from 39 samples (12 CHD, 9 NCHD, and 18 HT) was sequenced using the HiSeq platform (Illumina) and paired-end reads were generated. The raw data were collected using Readfq V8 to acquire clean data for subsequent analysis, producing an average of 540 million metagenomic jointed reads (12 Gb) per sample.

### Bioinformatics and Statistical Analyses

The reads of 16S rDNA were trimmed based on the cut-off of Q-value >20, missing bases >10%, and relative abundance <40%. The high-quality-paired reads were merged into a single tag based on the overlapping region using FLASH v1.2.11 (Magoč and Salzberg, 2011), with a minimum match length of 10 bp and 2% mismatch allowed in the overlapping region. Filtering was performed according to the protocols provided by the QIIME pipeline v1.9.1 (Caporaso et al., 2010). Pre-processed sequences were clustered at a 97% nucleotide sequence similarity level and taxonomic information was annotated using closed-reference operational taxonomic units (OTUs) against the reference database from Greengenes 13_8 (DeSantis et al., 2006).

Alpha/beta diversity and abundance were determined using Mothur v1.39.1 with default settings (Schloss et al., 2009). The Bray-Curtis distances were used to perform principal coordinate analysis (PCoA) using the R 3.4.5 packages of ade4 (Dray and Dufour, 2007) and vegan (Dixon, 2003). Relative abundance was calculated using normalization, in which the numbers of phyla and genera sequences were divided by the total number of sequences. The linear discriminant analysis effect size (LEfSe) method was used to assess differences among the microbial communities using a linear discriminant analysis (LDA) score threshold of two (Li and Yuan, 2005). Cytoscape v3.3.0 and its CoNet package were employed to generate network plots for co-occurrence and interactions (Faust and Raes, 2016). Physiological data, including the Spearman correlation matrix of the microbiota and the correspondence analysis (CA) of the patients' surveys, were also analyzed using the vegan package of R software.

For the metagenomic data of 39 samples, the DIAMOND software (v0.9.9) was used to blast Unigenes to a functional database with the parameter setting of blastp, and the best blast hit was used for subsequent analysis (Buchfink et al., 2015). The different functional profiles of microbial communities were processed and analyzed mainly using the KEGG database (Version 2018-01-01) (Kanehisa et al., 2014) and STAMP v2.1.3 (Parks et al., 2014).

## Results

### Summary of General Physiological Information

**Figure 1:**
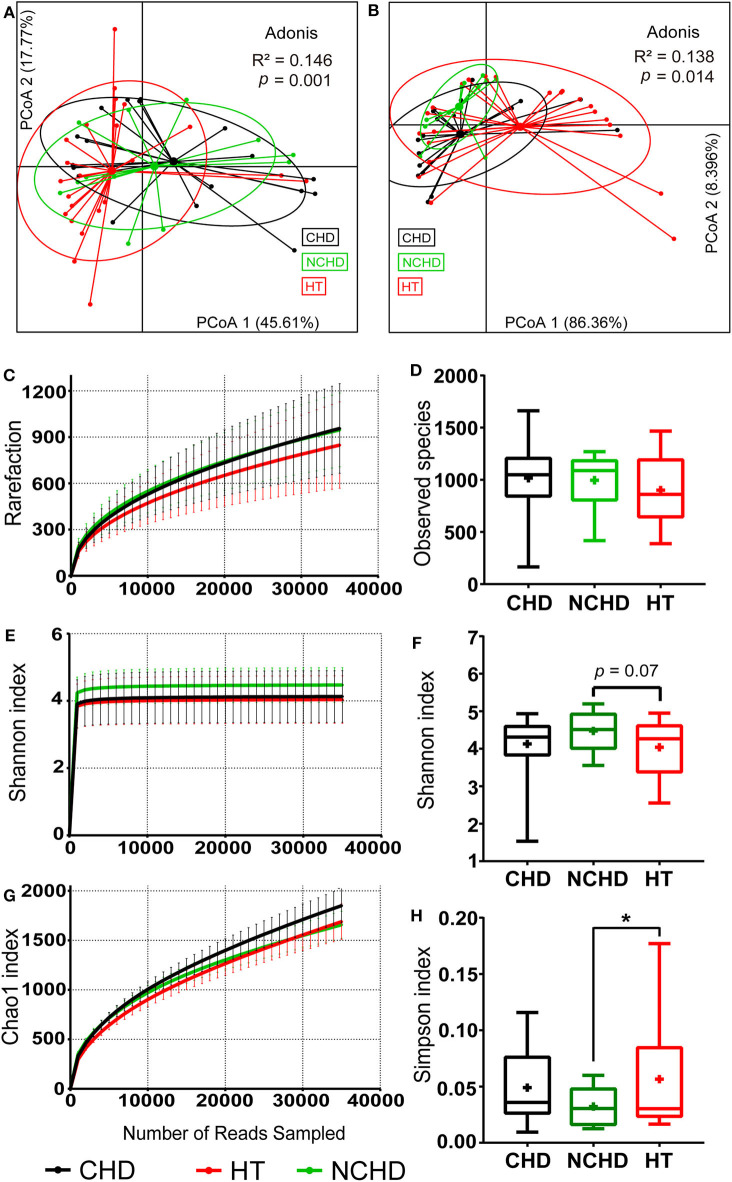
Physiological and microbial diversity in the three groups. **(A)** PCoA based on Bray–Curtis distances of the physiological indexes among the three groups in Table 1. **(B)** PCoA based on Bray–Curtis distances of the top 10 microbial phyla relative abundance among the three groups. **(C)** The number of observed operational taxonomic units. **(D)** Observed species. **(E)** Shannon curves for each group. **(F)** The Shannon index. **(G)** Chao1 curves for each group. **(H)** Simpson index Values are presented as mean ± SEM. Differences were calculated by one-way ANOVA and are denoted as: *p < 0.05.

Compared to the HT group, the HDL and APO-A1 levels of the CHD and NCHD groups were lower, while BUN/Cr, CRP, UA, and plasma and fecal LPS were significantly higher. WBC count, GLU levels, PLT count, betaine, creatinine, and l-carnitine were significantly higher in the CHD group than in the HT group. However, there were no significant differences in TC, TGs, LDL, RBC count, or TMAO levels associated with the examined gut microbiota (Table 1). Bray-Curtis PCoA revealed significant differences in physiological indices among the three groups (Figure 1A).

**Table 1:** General characteristics of the CHD, NCHD, and HT groups.

**Variables**	**CHD (*n* = 18)**	**NCHD (*n* = 12)**	**HT (*n* = 23)**
**Characteristics**			
Age (years)	53.3 ± 6.7^*^	53.0 ± 10.0^#^	41.5 ± 9.6
Sex (female/male)	0/18^*^	3/9^#^	13/10
Body Mass Index (kg/cm^2^)	26.5 ± 5.4	27.6 ± 5.4	25.2 ± 3.7
Systolic blood pressure (mmHg)	120.94 ± 16.48^*^	125.25 ± 20.65^#^	108.56 ± 14.12
Diastolic blood pressure (mmHg)	76.06 ± 12.61	84.75 ± 22.35	69.57 ± 10.55
Heart rate (bpm)	78.89 ± 11.43^*^	79.75 ± 20.94	67.78 ± 6.93
Smoking (yes/no)	8/10	4/8	5/18
Alcohol (yes/no)	2/16	3/9	2/21
Family medical history (yes/no/unknown)	9/6/3^*^	4/3/5	9/7/7
Occupation (herdsman/staff/unemployed)	4/11/3	4/6/2	9/5/9
Geographical locations(<3,500 m/≧3,500 m)	12/6	7/5	14/9
**Laboratory data**			
TC (mmol/L)	4.17 ± 1.12	4.36 ± 0.57	4.54 ± 0.81
TG (mmol/L)	1.50 ± 0.92	1.60 ± 0.87	1.27 ± 0.58
HDL (mmol/L)	1.03 ± 0.30^*^	0.98 ± 0.12^#^	1.18 ± 0.20
LDL (mmol/L)	2.30 ± 1.11	3.46 ± 0.68	3.29 ± 0.91
APO-A1 (g/L)	0.95 ± 0.22^**^	1.00 ± 0.15^#^	1.16 ± 0.14
APO-B (g/L)	0.68 ± 0.22	0.74 ± 0.18	0.72 ± 0.20
LP(a) (mg/L)	339.33 ± 216.48	328.25 ± 243.58	235.82 ± 199.06
BUN/Cr	0.077 ± 0.021^*^	0.080 ± 0.021^#^	0.062 ± 0.018
UA (μmol/L)	367.33 ± 81.58^**^	370.25 ± 82.95^##^	291.50 ± 66.04
GLU (mmol/L)	5.49 ± 0.97^*^	5.66 ± 1.64	4.76 ± 0.60
Hcy (μmol/L)	15.50 ± 6.61	11.18 ± 2.27	14.60 ± 8.81
CRP (mg/L)	32.29 ± 47.35^**^^▴^	5.88 ± 2.38	4.86 ± 1.56
WBC (10^9^/L)	9.96 ± 5.38^*^	6.81 ± 1.50	6.04 ± 1.46
RBC (10^12^/L)	5.16 ± 0.78	5.29 ± 0.60	5.19 ± 0.45
HGB (g/L)	159.33 ± 21.22	155.67 ± 19.28	151.43 ± 21.48
HCT (%)	47.83 ± 5.64	47.08 ± 5.05	46.22 ± 5.62
PLT (10^9^/L)	166.28 ± 47.38^**^	195.92 ± 62.17	219.65 ± 62.33
Choline (μmol/L)	10.97 ± 2.94	12.33 ± 3.22^##^	9.39 ± 2.14
Betaine (μmol/L)	62.15 ± 22.28^*^	57.99 ± 19.05	47.48 ± 16.86
TMAO (μmol/L)	5.17 ± 6.23	7.69 ± 5.69	4.08 ± 4.65
Creatinine (μmol/L)	95.10 ± 38.26^*^	89.79 ± 19.83	75.27 ± 17.29
L-carnitine (μmol/L)	46.04 ± 9.10^*^	45.58 ± 6.63	39.30 ± 10.00
Plasma LPS (pg/ml)	5.63 ± 0.72^*^^▴^	4.12 ± 0.63^##^	2.74 ± 0.67
Fecal LPS (pg/g)	38.05 ± 5.08^**^^▴▴^	21.04 ± 6.29^##^	10.68 ± 4.62

Values are presented as mean ± SD. All p-values are from the one-way ANOVA test or the Kruskal–Wallis test, except for sex, smoking, alcohol consumption, family history, profession, and geographical locations (χ^2^ tests).

* p < 0.05,

** p < 0.01, CHD vs. HT;

# p < 0.05,

## p < 0.01, NCHD vs. HT;

▴ p < 0.05,

▴▴ *p < 0.01, CHD vs. NCHD*.

### Taxonomic Alterations of the Gut Microbiome in the Three Groups

We defined the bacteria belonging to the top 10 phyla as core microbiota for beta diversity analysis (Astudillo-García et al., 2017) and conducted PCoA based on the Bray-Curtis dissimilarities of their relative abundance. We found that the sample centroids of the three groups were separated from each other and that the HT group was significantly separated from the other groups, suggesting that the variation in the microbial community composition may affect their association with CHD and NCHD (Figure 1B). OTUs clustered based on 97% similarity identified differences in OTU diversity between the three groups (Figure 1C). There was no difference of observed species between the three groups (Figure 1D). The NCHD group had a significantly lower Simpson index and higher Shannon index than the HT group while Chao1 index curve of the CHD group was higher than that of the other two groups (Figures 1E–H).

**Figure 2:**
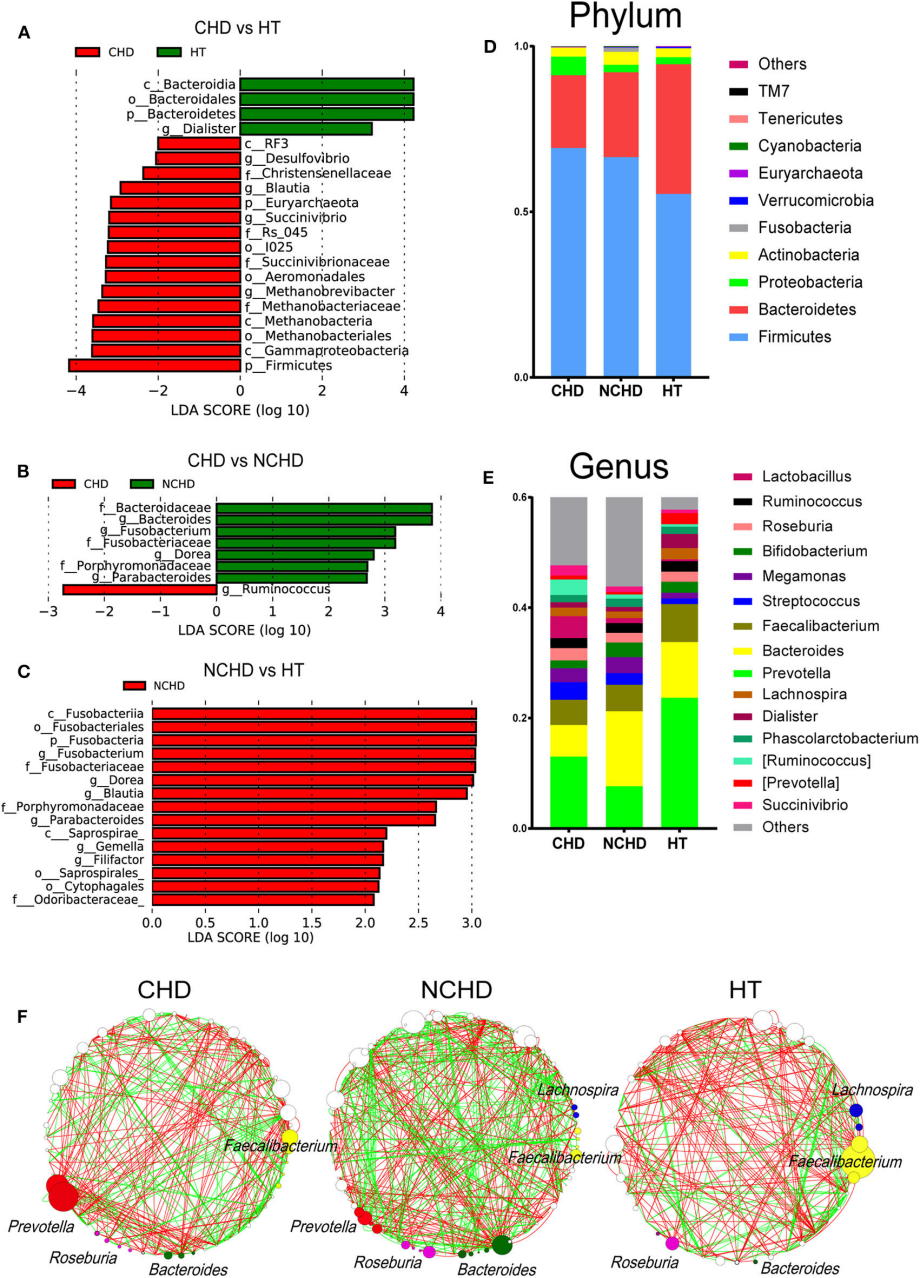
Taxonomic alterations in the three groups. Pairwise comparisons of CHD vs. HT **(A)**, CHD vs. NCHD **(B)**, and NCHD vs. HT **(C)** by the linear discriminant analysis effect size (LEfSe) method (LDA > 2, *p* < 0.05). Composition of gut bacterial communities at the phylum **(D)** and genus **(E)** levels. **(F)** Gut microbiota co-occurrence networks. Nodes represent operational taxonomic units, and the node sizes indicate different abundances. Links between the nodes represent significant Spearman correlations between the abundances of two operational taxonomic units; correlations >0.4 or lower than −0.4 were visualized. The color of the line reflects the direction (green: positive; red: negative).

The abundance of the *Dialister* genus and Bacteroidia class in the HT group was significantly higher than that in the CHD group (Figure 2A), while *Blautia, Desulfovibrio*, and *Succinivibrio* were higher in the CHD group. A higher abundance of the *Ruminococcus* genus was found in the CHD group than in the NCHD group (Figure 2B). In addition, higher LDA scores in the *Fusobacterium, Dorea, Blautia, Parabacteroides, Gemella*, and *Filifactor* genera were found in the NCHD group than in the HT group (Figure 2C). The abundance of Firmicutes in the HT group (55.4%) was significantly lower than that in the CHD (69.2%) and NCHD groups (66.5%). The abundance of Bacteroidetes in the HT group (39.1%) was significantly higher than that in the CHD (22.0%) and NCHD groups (25.6%), revealing a lower Firmicutes/Bacteroidetes (F/B) ratio in the HT group (Figure 2D, Table S2), although this was not statistically significant. At the genus level, the gut microbiota of the three groups was dominated by *Prevotella* (16.4%), *Bacteroides* (9.4%), and *Faecalibacterium* (5.6%). The HT group had a higher abundance of *Prevotella* (23.7%) and *Dialister* genera (2.6%) in comparison to the CHD (13 and 0.9%) and NCHD (7.6 and 0.8%) groups (Figure 2E, Table S3).

We found that the network structures in some patients were more complex, as the NCHD group had the most nodes and links, while the HT group had the least (Figure 2F). The genus *Faecalibacterium* was the most abundant network of the HT group, with lower abundances in the CHD and NCHD groups. In particular, *Prevotella* was the most abundant genus in the CHD group and *Bacteroides* was most frequently observed in the NCHD group, but these were barely detectable in the HT group. These three genera were more negatively correlated with the OTUs of other genera.

### Functional Changes of the Gut Microbiome in the Three Groups

**Figure 3:**
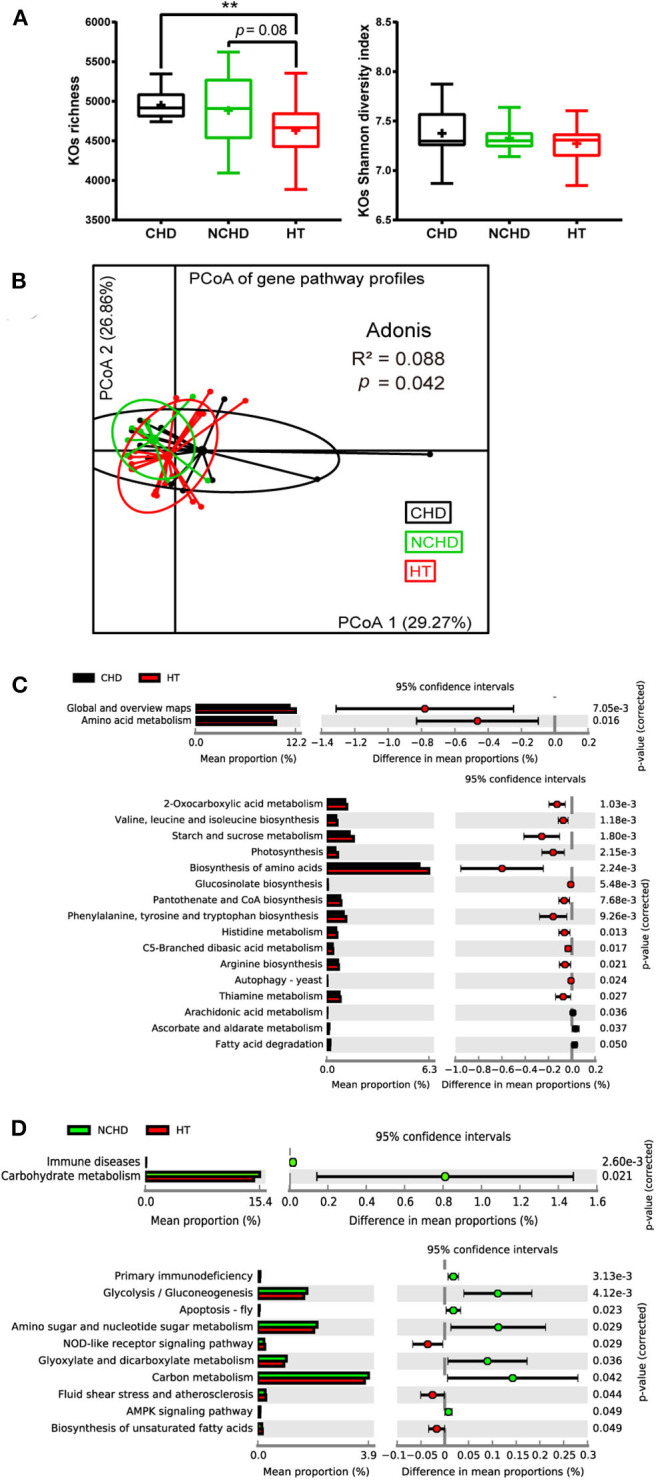
Functional variations of the gut microbiome between the three groups. **(A)** Boxplot of the KEGG orthology (KO) richness and KO Shannon diversity index. Differences were assessed by the Kruskal–Wallis test and are denoted as: **p < 0.01. **(B)** Dissimilarities in the level-3 functional profiles of gut microbiota among the three groups based on the Bray–Curtis distance derived from the relative abundance of genes using principal coordinate analysis. Level-2 and level-3 KEGG pathways were differentially represented between the CHD and HT **(C)** as well as NCHD and HT **(D)**. Differences were assessed by the Welch *t*-test and are denoted as the corrected *p*-value.

To gain an insight into the functional changes associated with the patients' gut microbiome, metagenomic sequencing data were processed and the relative abundance of the KEGG orthology (KO) and gene pathways were obtained. The richness of KOs, defined as the number of KOs detected within each subject, was significantly higher in the CHD group than in the HT group, while they were not significantly different in terms of KO Shannon diversity (Figure 3A). Functional similarities in gut microbiota were assessed by PCoA based on the Bray–Curtis distance derived from the relative abundance of gene enrichment in each pathway using Adonis analysis. The functions of the three groups were also different, as their gut microbiota community structures varied (Figure 3B).

We performed Welch *t*-tests using STAMP to compare the level-2 and level-3 KEGG pathways between the groups. Compared with the HT group, two level-2 pathways, including amino acid metabolism and global and overview maps, and 13 level-3 pathways, such as valine, leucine, and isoleucine biosynthesis, starch and sucrose metabolism, 2-oxocarboxylic acid metabolism, and thiamine metabolism, were decreased, while three level-3 pathways, including ascorbate and aldarate metabolism, arachidonic acid metabolism, and fatty acid degradation, were increased in the CHD group (Figure 3C). Three level-3 pathways, including the NOD-like receptor signaling pathway, fluid shear stress and atherosclerosis, and biosynthesis of unsaturated fatty acids, were decreased in the NCHD group compared with the HT group, while some pathways increased in the NCHD group compared with the HT group, including immune diseases and carbohydrate metabolism, which are level-2 pathways, and glycolysis/gluconeogenesis, glyoxylate metabolism, and dicarboxylate metabolism, which are among the seven level-3 pathways that were increased (Figure 3D).

### Correlation Between Physiological Indexes, Changes in Bacterial Taxa, and Microbial Functions

**Figure 4:**
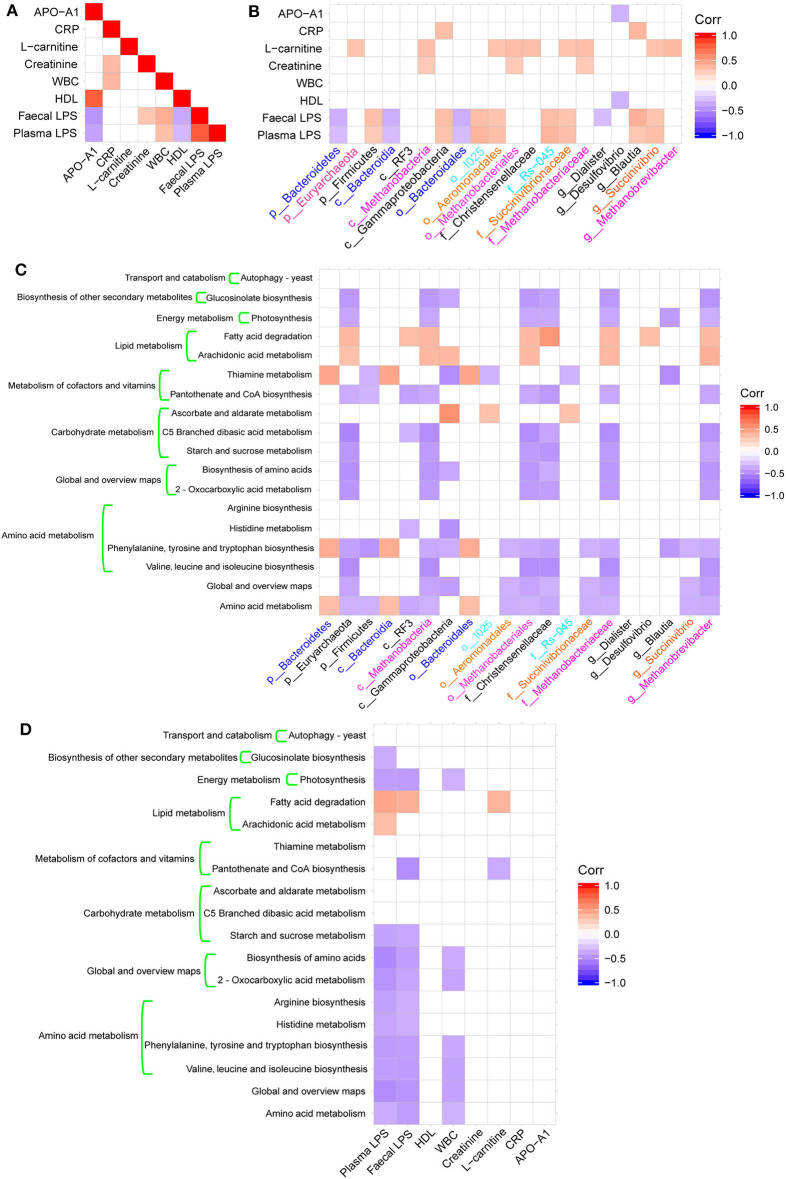
Heatmaps of Spearman's correlations. **(A)** Heatmap of correlations between altered physiological indices. **(B)** Heatmaps of correlations between altered microbial taxa and physiological indices. **(C)** Heatmap of correlations between altered microbial taxa and altered KEGG pathways. **(D)** Heatmap of correlations between physiological indexes and altered KEGG pathways.

We calculated the Spearman's correlation coefficients between some physiological indexes that were significantly different between groups (Table 1), bacterial taxa changes, and microbial functions (Figures 4A–D). The fecal and plasma LPS contents showed significant positive correlations with the presence of WBC (Figure 4A) and the abundance of Firmicutes, Gammaproteobacteria, *Blautia*, and *Succinivibrio* (Figure 4B). In contrast, the fecal and plasma LPS contents showed negative correlations with APO-A1, HDL (Figure 4A), and with abundance of Bacteriodetes (Figure 4B). Additionally, there was a negative correlation between fecal LPS and abundance of *Diaister* (Figure 4B).

These bacterial taxa changes had significant correlations with KEGG pathways associated with metabolic functions, including amino acid metabolism, carbohydrate metabolism, metabolism of cofactors and vitamins, lipid metabolism, and energy metabolism (Figure 4C). Additionally, there was a significant negative correlation between amino acid metabolism and global and overview map pathways with plasma or fecal LPS and WBC (Figure 4D).

### Lifestyle Gradients in Tibetan Population

**Figure 5:**
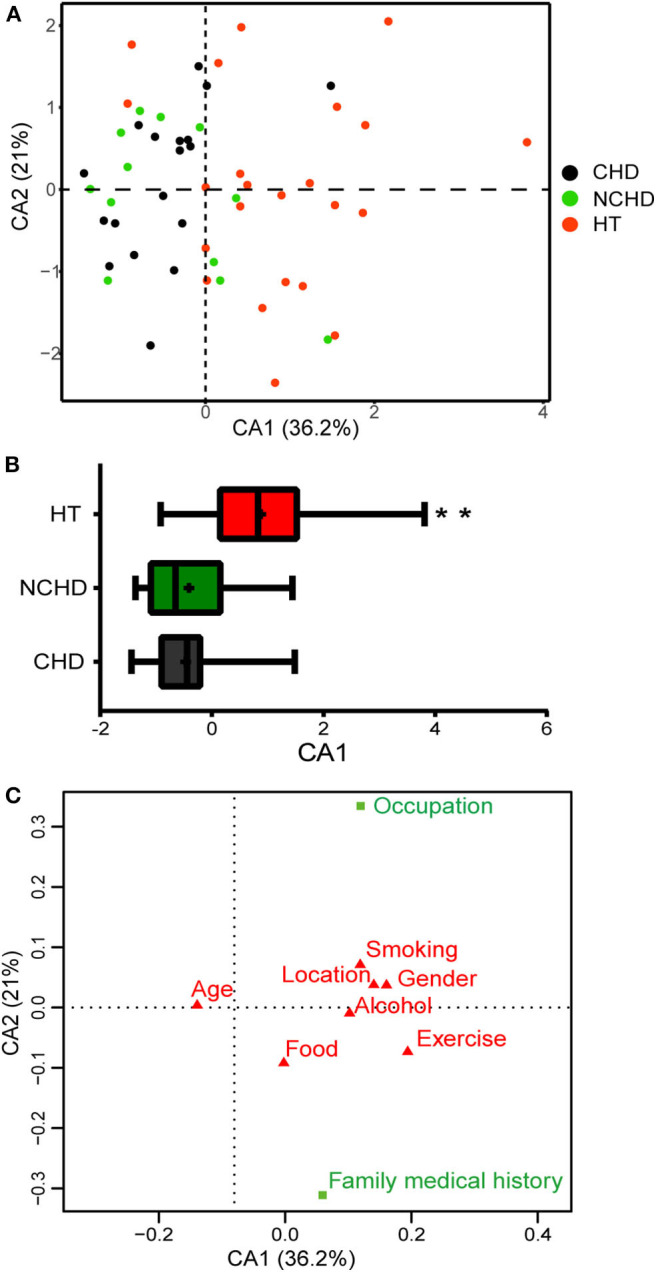
Correspondence analysis (CA) based on the lifestyle survey. **(A)** The first two dimensions of the CA and the amount of variation explained are shown. Each point represents an individual, and colors represent the groups. **(B)** Distribution of groups along the CA1 axis shows patterns of separation. Differences were assessed by the Kruskal-Wallis test and aredenoted as: **p < 0.01. **(C)** Factors in green (occupation and family medical history) are those that have greater Eigenvalues than expected and thus contribute the most to the top two dimensions in the CA.

We conducted surveys to assess the lifestyles of the participants in the three groups. The questionnaire covered exercise, food, smoking, alcohol consumption history, occupation, family medical history (FMH), and other factors. A correspondence analysis (CA) of the survey data revealed lifestyle differences among the three groups (Figure 5A). The first CA dimension (CA1) explained 36.2% of the variation and was significantly correlated with lifestyle differences. In CA1, the NCHD and CHD groups were different from the HT group (Figure 5B), indicating significant lifestyle differences among patients. Two variables, occupation, and FMH, contributed substantially to the first two CA dimensions and were significantly associated with cardiovascular and cerebrovascular diseases (Figure 5C). Therefore, we further explored the relationships between microbiota, occupation, and FMH.

**Figure 6:**
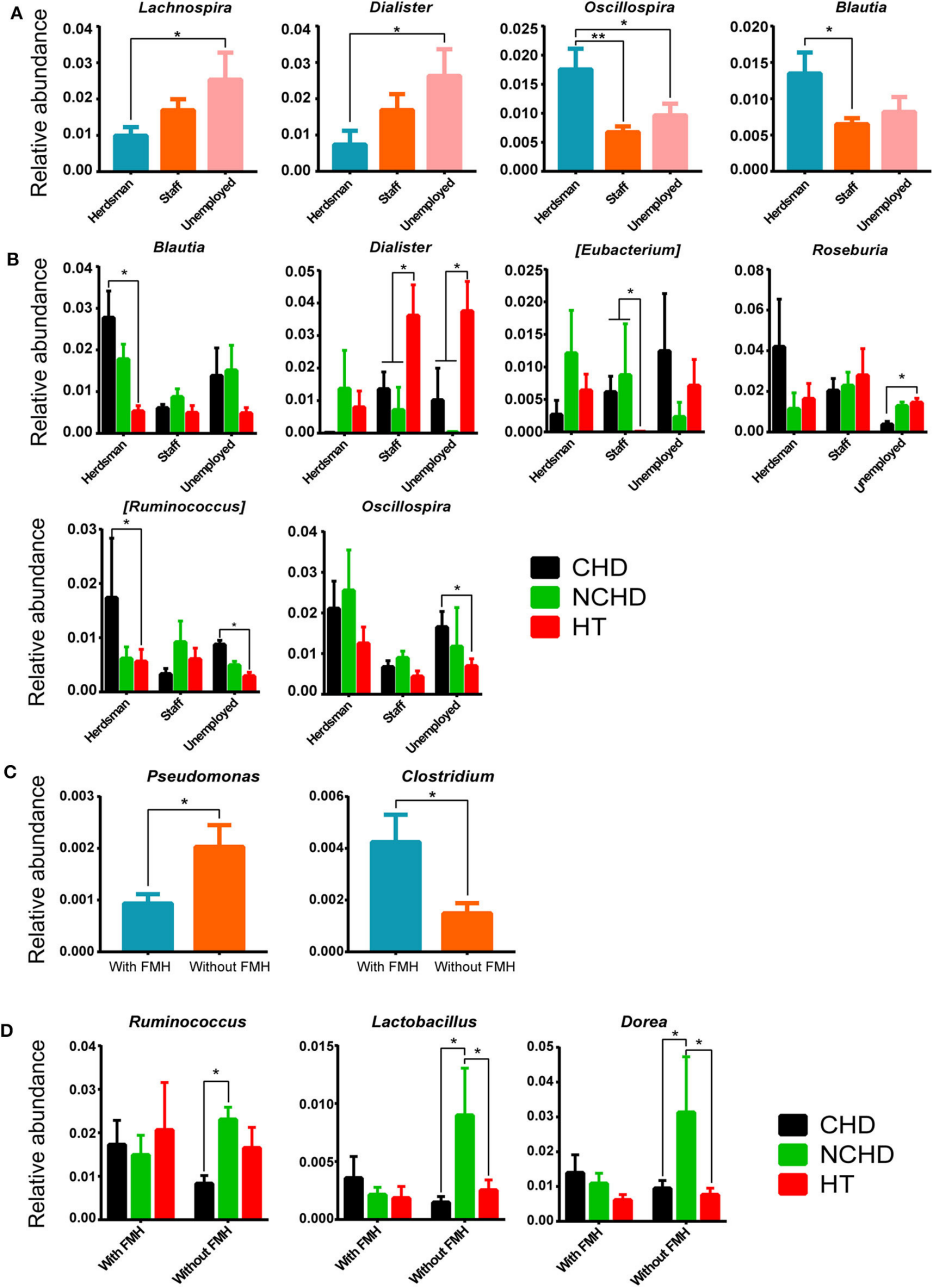
Effects of different occupations and family medical histories (FMHs) on gut microbiome. **(A)** Differences in bacterial relative abundance at the genus level among different occupations. **(B)** Bacterial relative abundance comparisons among the three groups and different careers at the genus level. **(C)** Differences in bacterial relative abundance at the genus level among people with and without a FMH of CHD. **(D)** Comparisons of bacterial relative abundance at the genus level among the three groups with/without a FMH of CHD. Values are presented as mean ± SEM. Differences were assessed by the Mann–Whitney U or Kruskal–Wallis tests and are denoted as follows: *corrected *p* < 0.05; **corrected *p* < 0.01.

A lower abundance of the *Lachnospira* and *Dialister* genera and a greater abundance of the *Oscillospira* and *Blautia* genera were found in herdsmen in comparison to other professions (Figure 6A), indicating that the tolerance threshold of herdsmen to alterations of gut microbiome was higher than others. Occupation played an important role in the CHD, NCHD, and HT groups, as we identified six bacterial genera with statistically significant differences, including *Blautia, Dialister, Eubacterium, Roseburia, Ruminococcus*, and *Oscillospira* (Figure 6B). A lower abundance of the *Pseudomonas* genus and a greater abundance of the *Clostridium* genus were found in people with a family medical history (FMH) of CHD than in those without (Figure 6C). We also found three bacterial genera, including *Ruminococcus, Lactobacillus*, and *Dorea*, with statistically significant differences between the three groups (Figure 6D).

## Discussion

Many studies have confirmed the role of TMAO in cardiovascular diseases, and plasma TMAO levels are the highest among patients with ischemic heart failure in some populations (Tang et al., 2013; Mafune et al., 2016). However, we found in this study that plasma TMAO levels in Tibetan patients with CHD were not higher than those in healthy subjects. Another study did not find evidence of a significant difference in plasma TMAO levels between Han patients with CHD and healthy individuals (Liu et al., 2019). Moreover, TMAO was not associated with coronary artery calcium levels or carotid intima-media thickness in black or white adults (Meyer et al., 2016). Low levels of TMAO are associated with cardiovascular disease and cardiometabolic risk in general (Arduini et al., 2019). In the present study, plasma levels of l-carnitine and betaine, which are related to choline metabolism, were significantly different in the CHD and NCHD groups compared to the HT group. Although these levels are also associated with the predicted risk of major adverse cardiac events, their prognostic value also depends on TMAO levels (Jie et al., 2017; He Y. et al., 2018). We found no significant difference between the CHD group and the HT group in terms of choline content and the abundance of TMA-producing bacteria (Table S4) (Zhu et al., 2016; Dalla Via et al., 2019). In addition, there was no difference in plasma TMAO levels between groups. These findings indicate that the TMAO pathogenic pathway may not play a role in Tibetan patients with CHD. The reasons why these bacterial taxa related to TMA production did not increase significantly in Tibetan patients with CHD should be explored in future studies.

LPS, also known as endotoxin, is a component of the outer membrane of gram-negative bacteria. LPS contains a hydrophobic lipid A component. The release of LPS from the bacterial membrane can induce systemic inflammation and sepsis. Thus, it can be used as a biomarker of CHD (Levels et al., 2001; Laugerette et al., 2010; Liljestrand et al., 2017). Alterations in the gut microbiome could lead to a significant increase in permeability of the intestinal barrier, allowing LPS to directly enter the circulatory system. On the other hand, an imbalance between beneficial and harmful bacteria could lead to the increased release of LPS by gram-negative bacteria, resulting in HDL levels that are insufficient for the transport of cholesterol, leading to the increased deposition of cholesterol in the vascular wall (Harris et al., 1990; Levels et al., 2005; Khan et al., 2018). We also observed that LPS levels in the CHD and NCHD groups were significantly higher than those in the HT group, which is consistent with previous studies in Chinese Han (Liu et al., 2019), Japanese (Mafune et al., 2016), and American (DiMeglio et al., 2019) patients with CHD. The same observation has been obtained in animal experiments (Tapping and Tobias, 2000). In addition, significant increases were observed in WBC count (which reached 9.96 ± 5.38 cells/L) and CRP levels in the CHD group. These results are indicative of a systemic low-level inflammatory response in Tibetan patients with CHD and the influence of the gut microbiome on physiological parameters.

We further found that the relative abundance of Bacteroidetes and *Dialister* in the CHD group was significantly lower than that in the HT group. Their relative abundances were negatively correlated with fecal LPS levels. Firmicutes, *Blautia*, and *Succinivibrio* were increased in abundance in the CHD and NCHD groups and significantly positively correlated with fecal and plasma LPS levels, indicating that these bacterial changes are closely related to intestinal disorders and inflammatory endotoxemia in Tibetan patients with CHD. *Blautia* is an important genus in the order Clostridiales, which is associated with increased obesity and cardiovascular disease (Zhu et al., 2018). The results of this Tibetan patient study are consistent with a previous report of higher fecal LPS levels in Japanese patients with coronary atherosclerosis compared to healthy individuals (Yoshida et al., 2018). Other studies in Russian (Vatanen et al., 2016), Finnish (Vatanen et al., 2016), Danish (Hersoug et al., 2018), and Spanish (Clemente-Postigo et al., 2013) subjects have also shown that LPS is associated with elevated levels of gram-positive bacteria (Firmicutes) and decreased levels of gram-negative bacteria (Bacteroidetes), but the specific genera were different from the Tibetans. For example, the relative abundance of *Bifidobacterium* was negatively correlated with LPS concentrations in Russians (Vatanen et al., 2016) and Spaniards (Clemente-Postigo et al., 2013). However, there was no such correlation among Tibetans, and there was no significant change in the abundance of this genus between Tibetan patients and healthy participants. It is worth noting that *Dialister*, which is a genus in the phylum Firmicutes, was also inversely proportional to fecal LPS. This genus has not been reported in other studies, so it may be a new target for the prevention and treatment of CHD in Tibetans by inhibiting intestinal and systemic inflammation. The collective findings show that the key bacterial groups and metabolic pathways in the same disease can be significantly different due to different ethnic and geographical environments. Thus, the pathogenesis can be diverse. Therefore, while an increase in LPS in patients with CHD is a common feature among different nationalities, the predominant bacteria producing LPS differ between nationalities and regions.

Tibetan people have a long history of a diet rich in red meat and yak butter (~90% animal fat). The monthly intake of beef and mutton by each adult exceeds 30 kg (Liu, 2012). The long-term intake of high levels of protein and fat may affect the amino acid and lipid metabolism functions of the gut microbiome. Compared with the HT group, the KO richness was significantly higher in the CHD group, consistent with the structural differences of each group. The pathways linked to amino acid metabolism and carbohydrate metabolism were decreased in the CHD group, while pathways linked to lipid metabolism were enhanced. Amino acid metabolism disorders might also lead to changes in intestinal permeability and inflammatory reactions, and *Dialister* species play a role in amino acid metabolism (Contreras et al., 2010). Bacteria are also involved in other metabolic pathways and are inhibited by the presence of LPS and LPS-containing bacteria, such as *Succinivibrionaceae*. Amino acids have a protective effect on intestinal injury, and decreased activity of amino acid metabolic pathways promotes intestinal permeability (He F. et al., 2018), which in turn increases the blood levels of LPS, leading to endotoxemia. The generation of heart disease is mostly related to changes in amino acid metabolism and enhanced lipid metabolism in Asian populations, including Chinese Han (Liu et al., 2019) and Japanese (Mafune et al., 2016). However, Tibetan patients have unique features in specific functional pathways. We observed alterations in fewer pathways in the above metabolic functions than previously described for Han (Jie et al., 2017; Liu et al., 2019) and Japanese (Mafune et al., 2016) patients.

There were more lines in the network structure of the CHD and NCHD groups than in the HT group, showing that the relationship and interactions between intestinal flora in patients with CHD were more complex than in HTs. A similar antagonistic relationship has been described between the two dominant groups, *Bacteroides* and *Prevotella*, in the microbiota of Asians and Americans (Ley, 2016). However, these two genera account for a large proportion of the microbiota network of both German patients with CHD and non-CHD individuals, and CHD reduces the proportion of *Prevotella* in the network (Kehrmann et al., 2019). It is noteworthy that the relationship between the two genera was weakened in the HT group, and the OTUs of the genus *Faecalibacterium* revealed it as the dominant bacterium, while other genera were more balanced. *Faecalibacterium* is considered an anti-inflammatory bacterium that is deficient in many metabolic disorders (Machiels et al., 2014; Lopez-Siles et al., 2016). The major genus in the microbiota network of CHD patients in the southern Han population has been identified (Hersoug et al., 2018). However, in the latter study, *Lachnospira* did not occupy a notable position in the network structure, while the genera *Ruminococcaceae* were important and may have been decreased in Dutch cardiovascular patients (Koopen et al., 2016). These results are quite different from our results, which showed specific gut microbial network interactions in Tibetan patients with CHD.

We also found that occupation and family history of CHD were the most important lifestyle and genetic factors associated with CHD in Tibetans. Over the past 40 years, rapid urbanization in China has led to Tibetans discovering jobs and settling in towns or cities, leading to the westernization of their gut microbiota (Li et al., 2018). The flora of urban herdsmen were changed with the degree of urbanization. Similar alterations have also occurred in the Himalayan populations of Nepal (Jha et al., 2018), and these changes in the gut microbiome are associated with CHD in Asian and American populations (Li and Zhao, 2015; Koopen et al., 2016; Tang et al., 2017). We speculated that the urbanization process reduced the tolerance of Tibetans to alterations in the gut microbiome. The influence of FMH of CHD has been widely accepted by researchers, and studies show that FMH of CHD predicts cardiovascular risk independent of established metabolic risk factors (Lloyd-Jones et al., 2004). The effects of genetic factors on CHD are complex and varied. The mechanism by which a host's genes promote the risk of CHD by modifying the composition and function of the gut microbiota in Tibetan subjects still needs to be explored.

In conclusion, we discovered risk biomarkers and a significant difference in the presence of the gut microbiome, including *Blautia, Dialister*, and *Succinivibrio* genera, between Tibetan patients with CHD and NCHD and healthy Tibetans. These findings suggest a combined effect from the gut microbial community. The pathology of CHD is accompanied by significantly increased plasma and fecal LPS, rather than TMAO. The bacterial alterations resulted in an appreciable reduction in intestinal amino acid metabolism pathways, and they were significantly related to LPS and HDL levels and numbers of WBCs, which may lead to low inflammatory coronary atherosclerosis. Finally, occupation and FMH of CHD were important risk factors in Tibetan individuals.

## Data Availability Statement

The measured 16S rDNA sequences have been uploaded to the National Center for Biotechnology Information using accession number: PRJNA550301. The metagenomics data are available from the corresponding author on reasonable request. Other data can be found in the article/Supplementary Material.

## Ethics Statement

The studies involving human participants were reviewed and approved by Ethics Committee of Qinghai Province Cardiovascular and Cerebrovascular Disease Specialist Hospital. The patients/participants provided their written informed consent to participate in this study.

## Author Contributions

FL and YZ conceived the study, directed the project, designed the experiments, interpreted the results, and wrote the manuscript. CF and LZ performed the computational and 16S rDNA microbiota analysis and wrote the results and methods. HH, YM, YT, and JF collected the samples and clinical data. YL supervised the fecal microbiota collection and detected the biochemistry parameters. SJ supervised the data analysis and revised the manuscript. TW revised the manuscript. All authors contributed to the article and approved the submitted version.

## Conflict of Interest

The authors declare that the research was conducted in the absence of any commercial or financial relationships that could be construed as a potential conflict of interest.
