# Supplementary material for: Alterations of Gut Microbiome in Tibetan Patients With Coronary Heart Disease
Source: Front Cell Infect Microbiol. 2020 Jul 23;10:373. doi: 10.3389/fcimb.2020.00373 (PMC7390946; doi:10.3389/fcimb.2020.00373)
Supplement: Supplementary file 1 [file Table_1.DOCX]

***Supplementary Material***

**Table S1. Brief diagnoses of patients in the CHD and NCHD groups.**

| No. | Examination result | Principal diagnosis | Secondary diagnosis |
| --- | --- | --- | --- |
| CHD-1 | ECG: Anterior wall myocardial infarction  ST-T wave inversions UCG: Left ventricular diastolic and systolic dysfunction(LVEF44%)  Regional wall motion abnormality  Atrial septal defect CTA: Left anterior descending (LAD) stenosis > 90% CAG: LAD stenosis > 90% | Coronary atherosclerotic heart disease Acute myocardial infarction | NYHA class II Congenital heart disease Type II atrial septal defect |
| CHD-4 | ECG: Inferior wall myocardial infarction  T wave inversions US(heart): Coronary artery stenting (LAD stenosis > 90%)  Left heart enlargement  Regional wall motion abnormality  Decreased cardiac function  ABP: Abnormal diastolic pressure | Coronary atherosclerotic heart disease Acute inferior myocardial infarction | Old myocardial infarction NYHA class I Postoperative status of stent implantation |
| CHD-6 | ECG: Inferior and anterior wal lwall myocardial infarction  ST-T wave inversions US(heart): Left ventricular enlargement  Regional wall motion abnormality  Decreased cardiac function  ABP: Hypertension Level II CAG: LAD and right coronary artery (RCA) stenosis > 90% | Coronary atherosclerotic heart disease Acute anterior myocardial infarction | NYHA class I Hypertension Level II (Extremely high risk) History of hypertension for 10 years |
| CHD-7 | ECG: Low Voltage of All-conducting QRS Wave Group  Acute inferior and right ventricular myocardial infarction US(heart): Left ventricular diastolic dysfunction | Coronary atherosclerotic heart disease Exertional angina | Old myocardial infarction Hypertriglyceridemia |
| CHD-8 | ECG: ST-T wave inversions  Inferior and posterior myocardial infarction  Low Voltage of Limb Guided QRS Wave Group  T wave inversions UCG: Regional wall motion abnormality  Decreased cardiac function  CDUS(vessels): Arteriosclerosis and plaque formation of the right   lower extremity (smooth blood flow) CAG: LAD stenosis >80%  Left circumflex coronary artery (LCX) stenosis 100% | Coronary atherosclerotic heart disease Acute myocardial infarction | NYHA class II |
| CHD-10 | ECG: Acute anterior myocardial infarction  ST-T wave inversions US(heart): Ejection fraction (EF) 48%  Left ventricular dysfunction  Regional wall motion abnormality CAG: LAD was completely occluded. | Coronary atherosclerotic heart disease Acute anterior myocardial infarction | NYHA class II |
| CHD-13 | ECG: Regional wall motion abnormality  UCG: Basal thickening of ventricular septum  Hydropericardium  Decreased cardiac function  ABP: Abnormal circadian rhythm of systolic and diastolic blood pressure CAG: Proximal LAD stenosis 80%, LCX stenosis 85%, RCA occlusion | Coronary atherosclerotic heart disease Acute inferior myocardial infarction | NYHA class I Hypertension Level II (Extremely high risk) History of hypertension for 15 years |
| CHD-14 | ECG: T wave inversions UCG: Left ventricular wall motion abnormalities  Pulmonary hypertension  Left ventricular dysfunction CAG: Proximal LAD stenosis 85%  Middle LCX stenosis 50%  Middle OMI stenosis 90%. | Coronary atherosclerotic heart disease Unstable angina pectoris | Hypertension Level III (Extremely high risk) |
| CHD-15 | UCG: Left ventricular diastolic dysfunction CDUS(abdomen): Chronic cholecystitis CDUS(vessels): Bilateral common carotid artery intima-media thickening  Plaque formation at bifurcation of left common carotid artery ABP: Increased blood pressure and abnormal circadian rhythm CAG: Left main coronary artery (LM) stenosis 25% Proximal LAD stenosis 75% RCA stenosis <50% Small branch stenosis < 25% | Coronary atherosclerotic heart disease Unstable angina pectoris | Hypertension Level III (Extremely high risk) Arrhythmia, Atrial premature beats Carotid atherosclerosis History of hypertension for 20 years |
| CHD-16 | ECG: Right ventricular wall myocardial infarction UCG: Left atrial enlargement  Left ventricular wall motion abnormalities  Left ventricular dysfunction (EF49%) CAG: Proximal and middle LAD stenosis 80%  LCX, RCA and posterior descending artery (PDA) had scattered plaques | Coronary atherosclerotic heart disease Unstable angina pectoris | NYHA class II Type II diabetes Hyperlipidemia History of diabetes mellitus for many years |
| CHD-17 | ECG: Inferior wall myocardial infarction  T wave inversions UCG: Regional wall motion abnormality  Left ventricular dysfunction  CDUS(vessels): Plaque formation at bifurcation of lateral common carotid artery  Right common carotid artery intima-media thickening CAG: Middle LAD stenosis 75%, Proximal LAD stenosis 60%,   Middle and distal LCX stenosis 60% ABP: Abnormal circadian rhythm of systolic and diastolic blood pressure | Coronary atherosclerotic heart disease Old inferior myocardial infarction Postoperative status of stent implantation | NYHA class III Carotid atherosclerosis Hyperlipidemia History of coronary heart disease for7 years. |
| CHD-19 | ECG: Roughly normal (after stenting) UCG: Left atrial enlargement  Pulmonary hypertension  Left ventricular diastolic dysfunction CAG: Proximal LAD stenosis 90% ABP: Abnormal circadian rhythm of systolic and diastolic blood pressure | Coronary atherosclerotic heart disease Postoperative status of stent implantation | Hypertension Level II (Extremely high risk) History of hypertension for 1.5 years |
| CHD-21 | ECG: ST-T wave inversions  Acute inferior myocardial infarction UCG: Left atrial enlargement  Regional wall motion abnormality  Left ventricular diastolic dysfunction CAG: Proximal RCA stenosis 50%,   Limited stenosis in 50% of PLA (Posterior collateral branch of  left circumflex coronary artery) openings, Slow LAD flow DCG: ST-T wave inversions | Coronary atherosclerotic heart disease Acute myocardial infarction | NYHA class II Slow coronary flow Hypertension Level III (Extremely high risk) High altitude polycythemia History of hypertension for 4 years |
| CHD-25 | ECG: Obvious sinus bradycardia  UCG: Left ventricular wall dyskinesia  Pulmonary hypertension CDUS(vessels): Bilateral common carotid artery intima-media thickening  Plaque formation at bifurcation of left common carotid artery CTA: Proximal RCA stenosis 50%  Proximal LDA had non-calcified plaque and stenosis > 70% | Coronary atherosclerotic heart disease Hypertension Level III (Extremely high risk) | Unstable angina pectoris Arrhythmia Sinus bradycardia Carotid atherosclerosis History of hypertension for 4 years |
| CHD-30 | UCG: Left and right ventricular enlargement  Regional wall motion abnormality  Left ventricular systolic dysfunction (EF40%) CTA: Middle RCA stenosis <50%  Vascular lumen at the second turning point of middle RCA stenosis >70%  Proximal LAD occlusion  Left ventricular enlargement  Anterior wall of left ventricle  Ventricular septum near apex  Apical myocardial perfusion defect DCG: ST-T wave inversions | Coronary atherosclerotic heart disease Ischemic cardiomyopathy | NYHA class II Old anterior intermural myocardial infarction History of hypertension for 2 years |
| CHD-31 | CAG: The first diagonal branch of LAD (D1) stenosis >70% ECG: First degree atrioventricular block  CT(Brain): Left radiation coronary lacunar infarction UCG: Left atrial enlargement  Left ventricular diastolic dysfunction (EF57%) US(Carotid artery): Right common carotid artery intima-media thickening  Plaque formation at bifurcation of left common carotid artery US(abdomen): Fatty liver, Gallstones ABP: Abnormal circadian rhythm of systolic and diastolic blood pressure DCG: ST-T wave inversions | Coronary atherosclerotic heart disease Unstable angina pectoris | Hypertension Level III (Extremely high risk) Lacunar infarction Carotid atherosclerosis History of hypertension for 7 years |
| CHD-32 | ECG: Acute anterior myocardial infarction  ST-T wave inversions  Complete left bundle branch block UCG: Left atrial enlargement  Left ventricular dysfunction  Regional wall motion abnormality DCG: Sinus rhythm  Frequent ventricular premature beats  ST-T wave inversions ABP: Abnormal circadian rhythm of systolic and diastolic blood pressure | Coronary atherosclerotic heart disease Acute anterior myocardial infarction | Arrhythmia Hypertension Level III (Extremely high risk) Secondary polycythemia History of hypertension for 5 years |
| CHD-33 | ECG: Acute inferior myocardial infarction CAG: RCA occlusion 100%. UCG: Left atrial enlargement  Left ventricular diastolic dysfunction   Left ventricular wall motion abnormalities DCG: Sinus rhythm, Occasional ventricular premature beats ABP: Abnormal circadian rhythm of systolic and diastolic blood pressure | Coronary atherosclerotic heart disease Postoperative status of stent implantation | Hypothyroidism Hypertension Level III (Extremely high risk) |
| NCHD-1 | ECG: Left ventricular hypertrophy  ST-T wave inversions CT(kidney): Left adrenal hyperplasia CTA: Proximal and middle RCA vascular wall thickening, distal localized lumen dilatation  Proximal LAD vascular wall thickening US(Vessels): Plaque formation at bifurcation of lateral common carotid artery  Left common carotid artery intima-media thickening UCG: Left heart and right atrial enlargement  Pulmonary hypertension  Left ventricular dysfunction (LEVF 29%) DCG: Sinus rhythm  ST-T wave inversions ABP: Abnormal circadian rhythm of systolic and diastolic blood pressure | Secondary hypertension | Ischemic cardiomyopathy NYHA class II Pulmonary hypertension Carotid atherosclerosis History of hypertension for 5 years |
| NCHD-2 | ECG: Normal X-ray: Narrowing of cervical intervertebral space  Osteophyte formation of joint US(abdomen): Fatty liver DCG: Sinus rhythm  Atrial premature beats ABP: Abnormal circadian rhythm of systolic and diastolic blood pressure | Cervical spondylopathy | Sinus rhythm Atrial premature beats Fatty liver History of hypertension for 2 years |
| NCHD-6 | CTA: Proximal LAD vascular wall thickening ECG: Frequent atrial premature beats  T wave inversions UCG: Left ventricular wall dyskinesia  Left ventricular diastolic dysfunction (LVEF 59%) US(Vessels): The plaque formed at the bifurcation of the left common carotid artery  Right common carotid artery to intracranial artery intima-media thickening | Unstable angina pectoris | Carotid atherosclerosis |
| NCHD-7 | ECG: Rapid atrial fibrillation  T wave inversions UCG: Whole heart enlargement  Left ventricular wall dyskinesia  Atrial septal defect  Pulmonary hypertension  Left ventricular dysfunction (LVEF 34%) US(Vessels): Left common carotid artery intima-media thickening  Right common carotid artery intima-media thickening with plaque formation DCG: Sinus rhythm  Occasional atrial premature beats ABP: Abnormal circadian rhythm of systolic and diastolic blood pressure | Hypertension Level III (Extremely high risk) | Arrhythmia Paroxysmal atrial fibrillation NYHA class III Atrial septal defect History of hypertension for 3 years |
| NCHD-8 | ECG: Sinus bradycardia  T wave inversions UCG: No abnormalities in cardiac structure and blood flow were found  Left ventricular function is normal | Paroxysmal supra ventricular tachycardia |  |
| NCHD-9 | ECG: Sinus bradycardia  ST-T wave inversions DCG: Sinus bradycardia  ST-T wave inversions UCG: No abnormalities in cardiac structure and blood flow were found  LVEF 65% ABP: Circadian increase and abnormal circadian rhythm of systolic and diastolic blood   pressure | Hypertension Level III (Extremely high risk) History of hypertension for 2 years | Hypertriglyceridemia |
| NCHD-13 | ECG: Rapid atrial fibrillation  T wave inversions UCG: Whole heart enlargement  Left ventricular wall thickening  Pulmonary hypertension  Left ventricular dysfunction (LVEF 38%) ABP: Circadian increase and abnormal circadian rhythm of systolic and diastolic blood   pressure DCG: Atrial fibrillation  T wave inversions US(Vessels): Bilateral common carotid artery intima-media thickening  Plaque formation in bilateral internal carotid artery, left common carotid   artery and external carotid artery | Acute exacerbation of chronic cardiac insufficiency | Hypertension Level III (Extremely high risk) Arrhythmia Atrial fibrillation Carotid atherosclerosis Hyperuricemia History of hypertension for 6 years |
| NCHD-15 | DCG: Sinus rhythm  Atrial premature beats  Short paroxysmal atrial tachycardia,  Partial T wave inversions UCG: Normal blood flow in cardiac structure  Left ventricular function was normal (LVEF64%) US(abdomen): Fatty liver  Chronic cholecystitis US(Vessels): Bilateral carotid artery blood flow patency ABP: Circadian increase and abnormal circadian rhythm of systolic and diastolic blood pressure | Hypertension Level III (Extremely high risk) History of hypertension for 1 year | Atrial premature beats Preventricular contraction Paroxysmal atrial tachycardia, Hypothyroidism Fatty liver Chronic cholecystitis |
| NCHD-17 | ECG: I degree atrioventricular block  Occasional ventricular premature beats  ST-T wave inversions CT(Cranium): Bilateral subcortical frontal parietal lobe  Radioactive Coronary Lacunar Cerebral Infarction  Mild brain atrophy UCG: Left atrial enlargement  Left ventricular diastolic dysfunction (LVEF 57%) ECT: Limited non-calcified plaque formation in the proximal segment of the right coronary artery, Vessels stenosis < 50%. ABP: Circadian increase and abnormal circadian rhythm of systolic and diastolic blood pressure DCG: Sinus rhythm  Short paroxysmal atrial tachycardia  Ventricular premature beat | Hypertension Level III (Extremely high risk) History of hypertension for 1 year | Arrhythmia Ventricular premature beat Paroxysmal atrial tachycardia Atherosclerosis Hyperlipidemia Lacunar infarction Cerebral atrophy |
| NCHD-23 | ECG: ST wave low pressure CTA: No atherosclerotic plaques and stenosis were found in all coronary arteries UCG: Left ventricular diastolic dysfunction US(Vessels): Intima-media thickening at bifurcation of left common carotid artery US(abdomen): Fatty liver  Right lobe cyst of liver | Coronary atherosclerosis | Carotid atherosclerosis Fatty liver Hepatic cyst Chronic viral hepatitis B |
| NCHD-27 | ECG: Incomplete right bundle branch block CTA: No atherosclerotic plaques and stenosis were found in all coronary arteries  Scattered plaque at RCA orifice of right coronary artery UCG: Mild enlargement of left atrium  Left ventricular function is normal US(abdomen): Fatty liver DCG: Sinus rhythm  Occasional atrial premature beats | Arrhythmia | Atrial premature contraction Fatty liver |
| NCHD-28 | ECG: ST-T wave inversions DCG: Sinus rhythm  ST-T wave inversions UCG: No abnormalities in cardiac structure and blood flow  Left ventricular function is normal ABP: Circadian increase and abnormal circadian rhythm of systolic and diastolic blood pressure | Hypertension Level III (Extremely high risk) | Type II diabetes mellitus  History of diabetes mellitus for 4 years |

Note:

| ECG | Electrocardiogram |
| --- | --- |
| UCG | Echocardiography |
| CTA | Computed tomography coronary angiography |
| CAG | Coronary arteriography |
| STENT | Stent implantation |
| US | Ultrasound |
| DCG | Dynamic ECG |
| ABP | Ambulatory blood pressure |
| CDUS | Color doppler ultrasound |
| CT | Computed Tomography |
| ECT | Emission Computed Tomography |

**Table S2. Major phyla (top10) differences among the three groups.**

| Major Phylum | Relative abundance | | | Kruskal-Wallis *p* value | Mann-Whitney U *p* value | | |
| --- | --- | --- | --- | --- | --- | --- | --- |
|  | CHD | NCHD | HT |  | CHD VS NCHD | CHD VS HT | NCHD VS HT |
| Firmicutes | 0.6924 | 0.6652 | 0.5542 | 0.070 | 0.330 | **0.029** | 0.198 |
| Bacteroidetes | 0.2197 | 0.2561 | 0.3911 | **0.033** | 0.162 | **0.022** | **0.049** |
| Proteobacteria | 0.0566 | 0.0224 | 0.0217 | 0.073 | **0.047** | 0.066 | 0.466 |
| Actinobacteria | 0.0274 | 0.0395 | 0.0261 | 0.443 | 0.253 | 0.958 | 0.251 |
| Fusobacteria | 0 | 0.0126 | 0.0013 | 0.092 | 0.064 | 0.976 | **0.048** |
| Verrucomicrobia | 0.0001 | 0.0011 | 0.004 | 0.098 | 0.053 | **0.050** | 0.909 |
| Euryarchaeota | 0.0021 | 0.0009 | 0.0006 | **0.023** | 0.480 | **0.043** | **0.008** |
| Cyanobacteria | 0.0006 | 0.0015 | 0.0002 | 0.090 | 0.832 | **0.042** | 0.118 |
| Tenericutes | 0.0006 | 0.0001 | 0.0002 | 0.798 | 0.587 | 0.605 | 0.712 |
| TM7 | 0.0001 | 0.0002 | 0.0001 | 0.623 | 0.816 | 0.446 | 0.404 |

**Table S3. Major genera (top 20) differences among the three groups.**

| Major Genus | Relative abundance | | | Kruskal-Wallis *p* value | Mann-Whitney U *p* value | | |
| --- | --- | --- | --- | --- | --- | --- | --- |
|  | CHD | NCHD | HT |  | CHD VS NCHD | CHD VS HT | NCHD VS HT |
| *Prevotella* | 0.13012 | 0.07633 | 0.23656 | 0.318 | 0.553 | 0.331 | 0.054 |
| *Bacteroides* | 0.05737 | 0.13599 | 0.10112 | 0.109 | **0.038** | 0.563 | 0.110 |
| *Faecalibacterium* | 0.04571 | 0.04792 | 0.06875 | 0.264 | 0.966 | 0.164 | 0.187 |
| *Streptococcus* | 0.03165 | 0.02127 | 0.0101 | 0.880 | 0.866 | 0.694 | 0.651 |
| *Megamonas* | 0.02537 | 0.02885 | 0.01062 | 0.973 | 0.764 | 0.937 | 0.931 |
| *Bifidobacterium* | 0.01419 | 0.02655 | 0.01986 | 0.575 | 0.310 | 0.401 | 0.835 |
| *Roseburia* | 0.02231 | 0.01728 | 0.01805 | 0.956 | 0.799 | 0.793 | 0.907 |
| *Ruminococcus* | 0.01826 | 0.01797 | 0.01916 | 0.169 | **0.038** | 0.958 | 0.175 |
| *Lactobacillus* | 0.03964 | 0.00874 | 0.00336 | 0.215 | 0.108 | 0.636 | 0.144 |
| *Lachnospira* | 0.01541 | 0.01229 | 0.02046 | 0.722 | 0.966 | 0.495 | 0.509 |
| *Dialister* | 0.00993 | 0.00812 | 0.02559 | **0.041** | 0.949 | **0.027** | **0.045** |
| *Phascolarctobacterium* | 0.01289 | 0.015 | 0.01299 | 0.297 | 0.385 | 0.252 | 0.204 |
| *[Ruminococcus]* | 0.02812 | 0.00744 | 0.00461 | 0.357 | 0.641 | 0.318 | 0.187 |
| *[Prevotella]* | 0.00724 | 0.00477 | 0.01997 | 0.466 | 0.806 | 0.189 | 0.607 |
| *Succinivibrio* | 0.01845 | 0.00955 | 0.00652 | **0.047** | 0.075 | **0.026** | 0.850 |
| *Oscillospira* | 0.0115 | 0.0149 | 0.00852 | 0.306 | 0.472 | 0.386 | 0.135 |
| *Coprococcus* | 0.00914 | 0.00887 | 0.01288 | 0.620 | 0.446 | 0.386 | 0.754 |
| *Dorea* | 0.01035 | 0.01618 | 0.008 | **0.019** | **0.047** | 0.655 | **0.004** |
| *Parabacteroides* | 0.0076 | 0.01172 | 0.01073 | **0.022** | **0.028** | 0.325 | **0.012** |
| *Blautia* | 0.01213 | 0.01277 | 0.005 | **0.002** | 0.352 | **0.017** | **0.001** |

**Table S4. Major TMA producers’ differences among the three groups.**

| Major TMA producers | Relative abundance | | | Kruskal-Wallis *p* value | Mann-Whitney U *p* value | | |
| --- | --- | --- | --- | --- | --- | --- | --- |
|  | CHD | NCHD | HT |  | CHD VS NCHD | CHD VS HT | NCHD VS HT |
| f__Lachnospiraceae | 0.2258074 | 0.2260056 | 0.1976513 | 0.358 | 0.271 | 0.599 | 0.187 |
| f__Enterobacteriaceae | 0.0201885 | 0.0051054 | 0.0025938 | 0.115 | 0.602 | 0.057 | 0.154 |
| f__Ruminococcaceae | 0.2565289 | 0.2736719 | 0.2321324 | 0.513 | 0.611 | 0.528 | 0.251 |
| *g__Streptococcus* | 0.0316454 | 0.0212698 | 0.0101003 | 0.880 | 0.866 | 0.694 | 0.651 |
| *g__Clostridium* | 0.0028478 | 0.0022991 | 0.0027317 | 0.222 | 0.703 | 0.093 | 0.297 |
